# Use of Water in Typhoid Fever

**Published:** 1875-08

**Authors:** 


### Use of Water in Typhoid Fever.

Dr. A. Luton, of Rheims, submits the patient to an *absolute* diet. The only drink permitted is water, which may be cooled with ice, and any quantity is allowed. At first the water is drunk with avidity, then with moderation, and at last with a certain degree of satiety. It is sometimes vomited at the commencement, but tolerance is soon established. Under its influence, the stools are at first quite abundant, then they become less frequent, are less fetid, and finally there is constipation.

The duration of the treatment is subordinate to the general progress of the disease, varying from four to eight days, taking the fever as it usually runs. In treating the *enteritis*, however, for which the remedy is especially intended, three or four days may suffice, after which the alimentation is gradually improved.

The theory of this treatment is easy to comprehend. It depends upon the fact of the rapid alteration of the alimentary substances, and especially of the sugars and feculæ in contact with the diseased surfaces, and the products which play the *role* of ferments, which they furnish. Acrid, acid, and putrid substances result from this alteration, and increase the inflammation of the stomach and intestines.

These decompositions may be artificially produced by immersing animal membranes, a piece of typhoid-fever intestine, for instance, in a saccharated fluid. Alcoholic fermentation immediately commences, and in regular course follow the acetic, lactic, or butyric, and putrid fermentations. These take place at the ordinary temperature; how much more rapid must they be in the diseased digestive passages where the temperature is so elevated!

By simply depriving the patient of food and sweetened drinks, this cause of irritation is suppressed, and the ferments are destroyed by inanition, their natural aliment being cut off.

The present method is applicable to the various cases of acute enteritis, and especially typhoid enteritis. In the hands of Dr. Luton the exclusive use of cold water as a drink, united with a rigorous diet, has become the best treatment of typhoid fever itself. The putridity, the subsequent adynemia, the visceral conjestions, the sloughs of the sacrum, and the fuligenous condition of the mouth, all cease, as if by enchantment, whatever may be the theory.

The indications which may arise in each ca e should be fulfillec. Thus, at the commencement, if there should be much gastric trouble, an emeto-cathartic should be prescribed; in the pseudo-intermittent stage, sulphate of quinine; a fatiguing cough is checked by bromide of potassium in cherry-laurel water. As the general condition of the patient improves, the diet may be gradually improved. Give at first milk in small quantities, then broths, and at last meats and wine, if no relaps occurs.—*Mouv. Méd. and Trib. Méd.—N. Y. Med. Journal.*

